# Supplementary figures and images for: Oxyresveratrol Possesses DNA Damaging Activity
Source: Molecules. 2020 Jun 1;25(11):2577. doi: 10.3390/molecules25112577 (PMC7321165; doi:10.3390/molecules25112577)

Oxy-Acetone-d6

PROTON.1jmu Acetone {C:\Bruker\TopSpin3.2p17} STGSSRADA 18

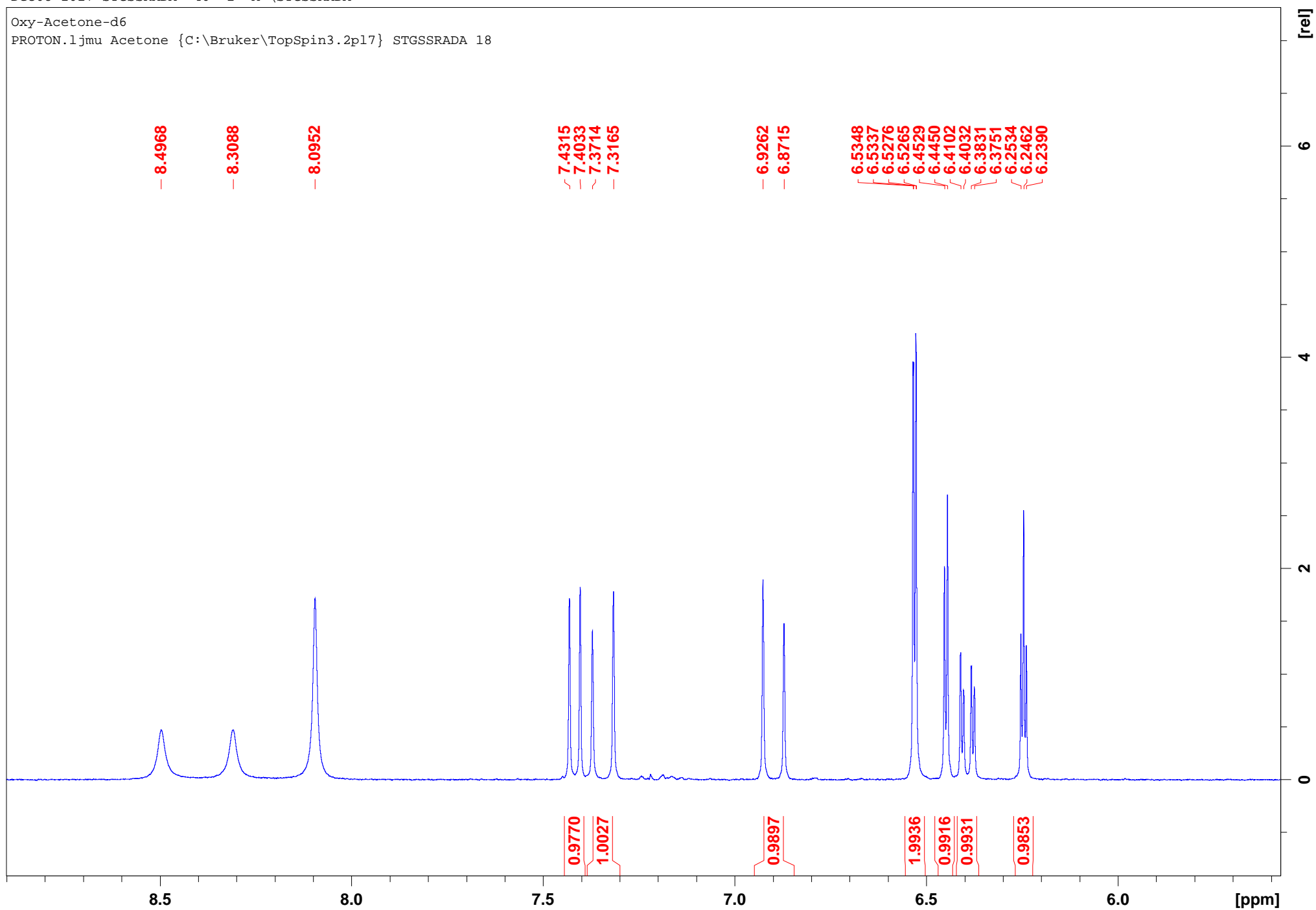

Supplement: Supplementary file 1 [file molecules-25-02577-s001.zip › Supplementary files/Figure S1.1H NMR Oxyresveratrol.pdf]

Oxyresveratrol

C13CPD Acetone {C:\Bruker\TopSpin3.2p17} STGSSRADA 4

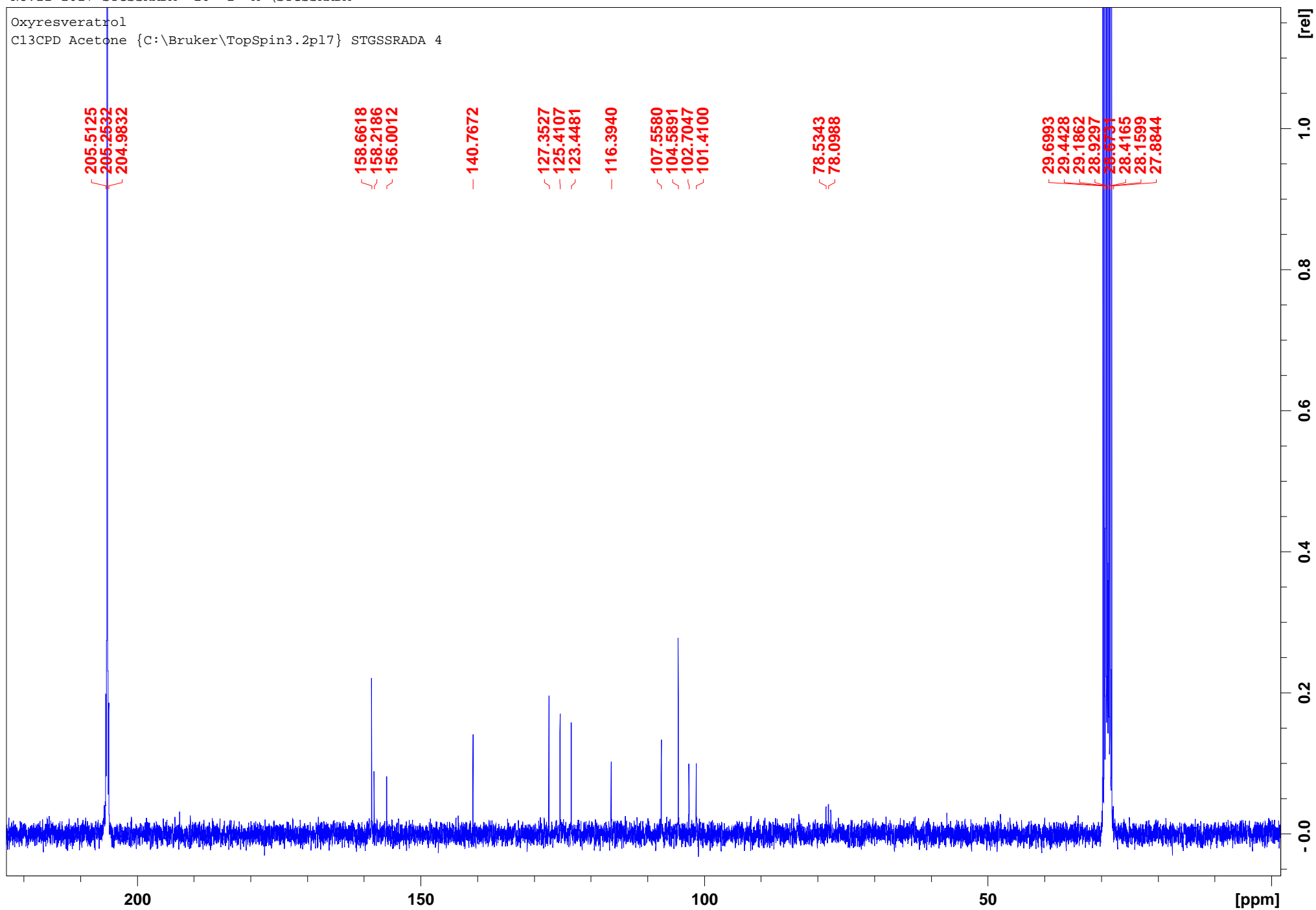

Supplement: Supplementary file 1 [file molecules-25-02577-s001.zip › Supplementary files/Figure S2.13C NMR Oxyresveratrol.pdf]

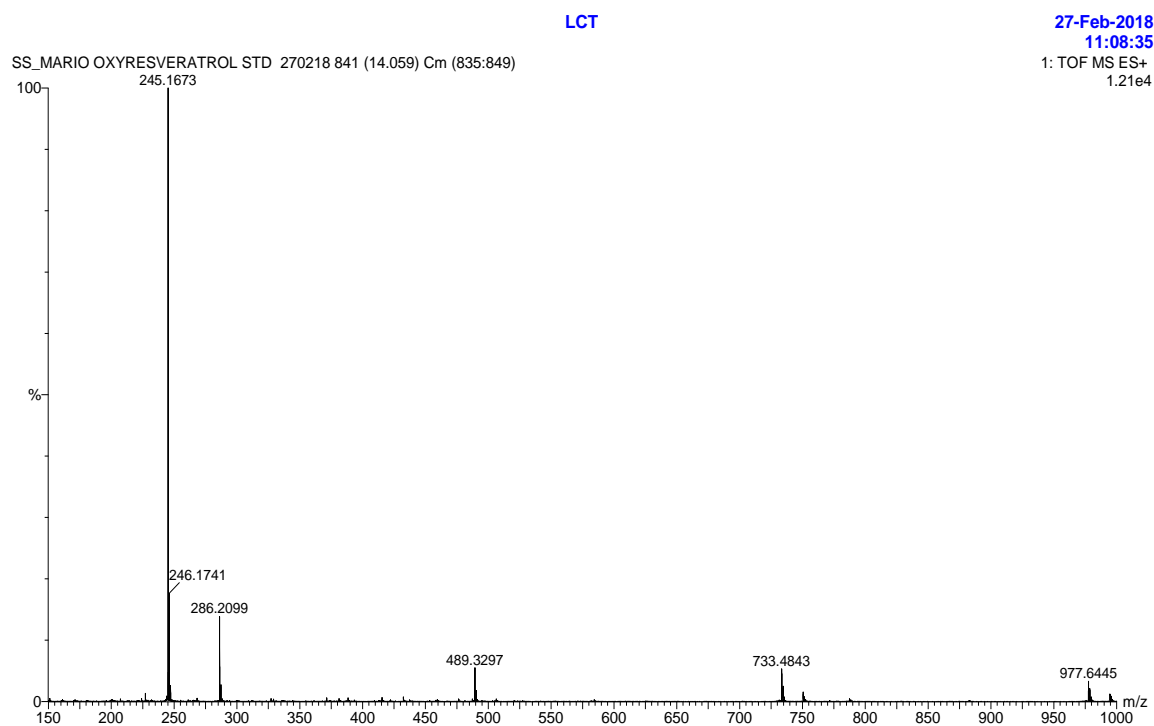

**Figure S3.** ESI-MS spectrum of standard oxyresveratrol

Supplement: Supplementary file 1 [file molecules-25-02577-s001.zip › Supplementary files/Figure S3. ESI-MS spectrum of standard oxyresveratrol.pdf]

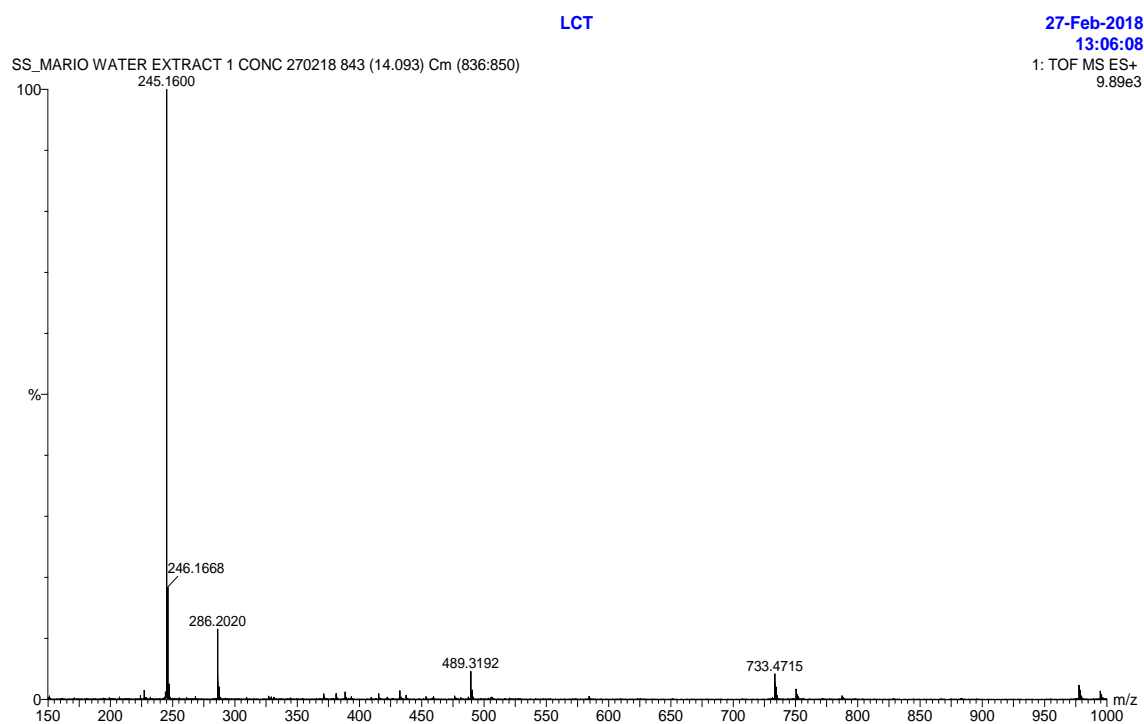

**Figure S4.** ESI-MS spectrum of the peak RT 14.0 in the water extract.

Supplement: Supplementary file 1 [file molecules-25-02577-s001.zip › Supplementary files/Figure S4. ESI-MS spectrum of the peak RT 14.0 in the water extract..docx.pdf]

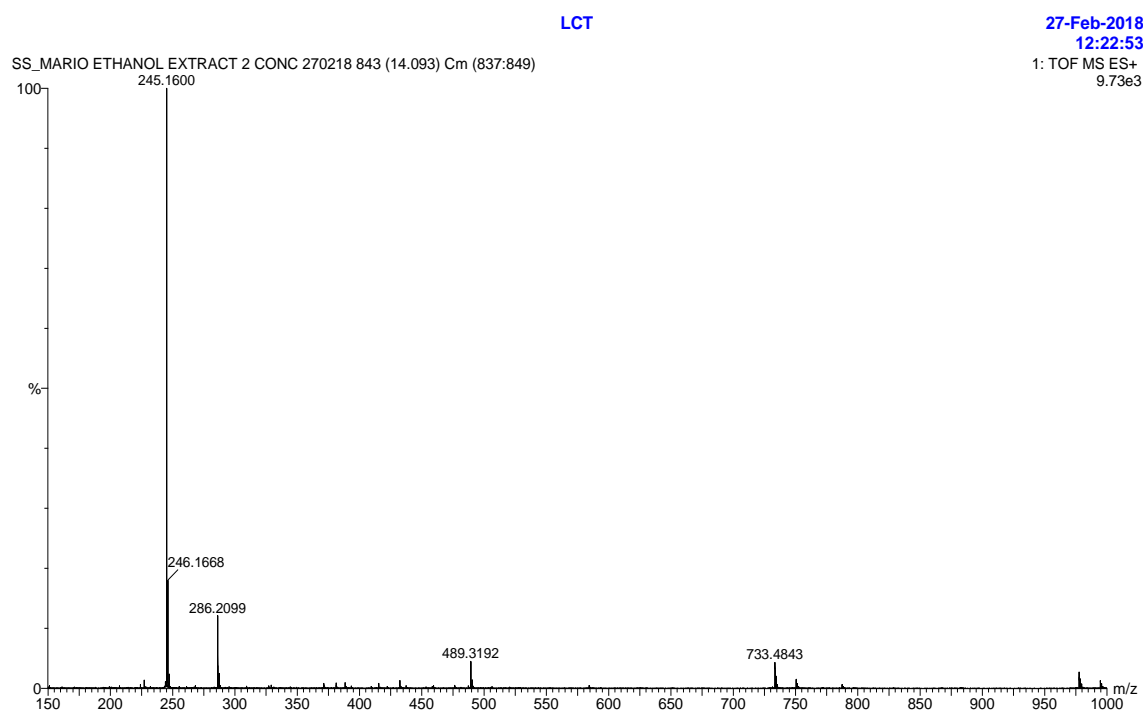

**Figure S5.** ESI-MS spectrum of the peak RT 14.0 in the ethanol extract.

Supplement: Supplementary file 1 [file molecules-25-02577-s001.zip › Supplementary files/Figure S5. ESI-MS spectrum of the peak RT 14.0 in the ethanol extract..pdf]

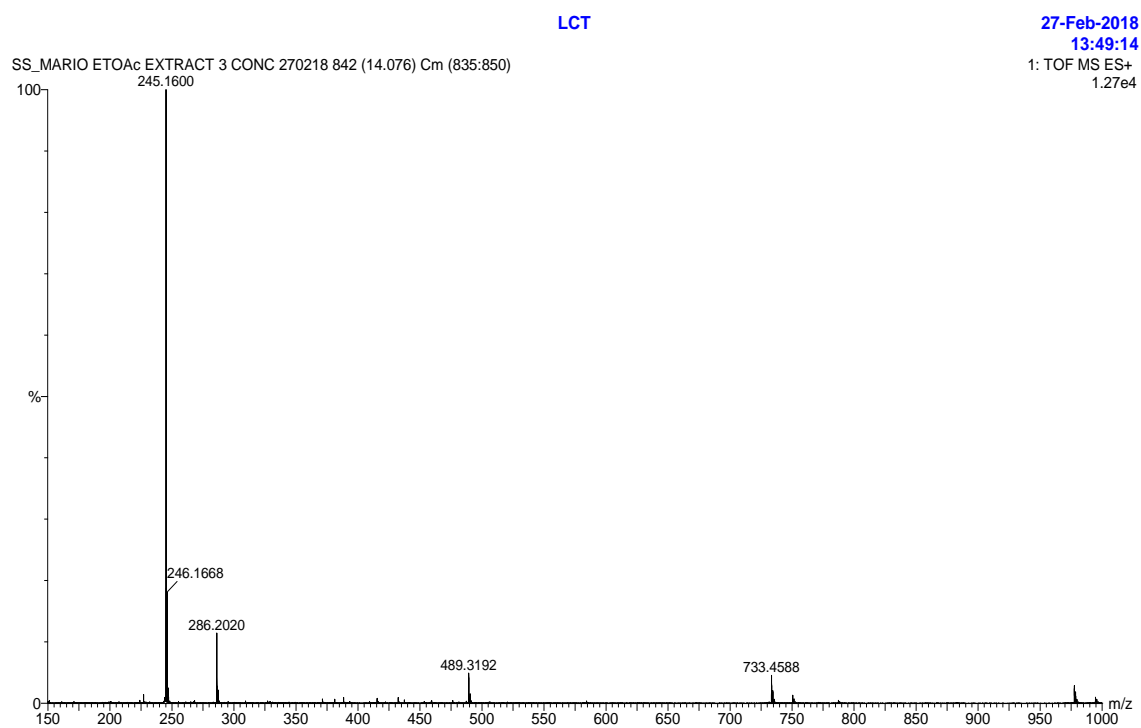

**Figure S6.** ESI-MS spectrum of the peak RT 14.0 in the ethyl acetate extract.

Supplement: Supplementary file 1 [file molecules-25-02577-s001.zip › Supplementary files/Figure S6. ESI-MS spectrum of the peak RT 14.0 in the ethyl acetate extract..pdf]
